# A Case of Bacteremia and Meningitis Associated with Piperacillin-Tazobactam Nonsusceptible, Ceftriaxone Susceptible *Escherichia coli* during *Strongyloides* Hyperinfection in an Immunocompromised Host

**DOI:** 10.1155/2017/8634717

**Published:** 2017-11-22

**Authors:** Santosh Dahal, Jeffrey Lederman, Jesse Berman, Marius Viseroi, Stephen Jesmajian

**Affiliations:** Department of Internal Medicine, Albert Einstein College of Medicine, Montefiore Medical Center, New Rochelle, NY 10801, USA

## Abstract

Strongyloidiasis is an emerging parasitic infection with intriguing epidemiology, presentation, and clinical management. We report a case of hyperinfection syndrome complicated by *E. coli* bacteremia and meningitis with one of the isolates showing a unique resistance pattern recently being recognized. This report describes the aspect of invasive bacterial infections in strongyloidiasis and highlights the unique susceptibility pattern of the *E. coli* isolate and the extreme caution required during the antibiotic therapy.

## 1. Introduction

Invasive bacterial infections, including meningitis by enteric and other commensal bacteria, are a known complication of severe *Strongyloides* spp. infection. Mortality rates as high as 60% and 68.5% have been reported for hyperinfection syndrome and disseminated infection, respectively [1]. Furthermore, Gram-negative bacillary meningitis by itself is a serious infection with a case fatality rate between 15 and 40% [2]. Eradication of the parasite is pivotal in the management, and it can be challenging in an immunocompromised host. Appropriate antibiotic therapy is the cornerstone in the management.

## 2. Case Presentation

The case is a 59-year-old Hispanic female born in Mexico with a medical history of insulin-dependent type 2 diabetes and rheumatoid arthritis on methotrexate, abatacept, and prednisone. Her prednisone dose was increased because of corneal transplant two days prior to admission. The patient was seen in the emergency department multiple times over the preceding month for abdominal pain, diarrhea, and vomiting and was treated with ciprofloxacin and metronidazole a week prior. She presented with lethargy, temperature 102°F, heart rate 121 bpm, and blood pressure 102/80 mmHg. Her Glasgow Coma Scale (GCS) score was 15/15, and meningeal signs were absent. The blood leukocyte count was 16,600 cells/mcL, serum sodium was 121 mmol/L, potassium was 3.4 mmol/L, chloride was 89 mmol/L, CO_2_ was 10 mmol/L, and blood sugar was 222 mg/dl. Arterial blood gas analysis showed triple acid-base disturbance with high anion gap metabolic acidosis, metabolic alkalosis, and respiratory alkalosis (pH 7.35, pCO_2_ 13, and HCO_3_ 7.1). Urine showed large ketones. CT scan of the abdomen showed possible colitis with thickening of the colon. She was started on insulin protocol after supplementing potassium. She was started on vancomycin 1.25 g every 12 hours (q12 hr) and piperacillin-tazobactam (PTZ) 3.37 g every 6 hours (q6 hr). Immunosuppressive agents were put on hold. She became more acidotic and tachypneic requiring intubation to prevent respiratory fatigue. On day 2 (D2), blood cultures grew Gram-negative bacilli (GNB). Suspicion of strongyloidiasis was high in the setting of Gram-negative bacteremia and a recent history of persistent GI symptoms in a patient from an endemic region. Single dose of ivermectin 200 micrograms/kg was given empirically. On D3, stool and sputum microscopy reported larvae of *Strongyloides* spp. GNB from blood culture was identified as *E. coli*, which was sensitive to cefazolin and piperacillin-tazobactam (PTZ) (Table 1). Hence, PTZ was continued.

She improved clinically, remained afebrile, and got extubated on D4, was started on oral diet on D5, and was moved out of ICU to regular floor on D6. Serology for *Strongyloides* was reported to be positive for immunoglobulin (Ig) A. On day 7, PTZ was switched with cephalexin 500 mg q6 hr. Early the next morning, she was found to have rapidly worsening mental status and was reintubated. Cephalexin was switched with cefepime 2 g q8 hr and ampicillin 2 g q4 hr. Lumbar puncture (LP) was done, and CSF showed GNB, WBC 330 cells/mcL with 98% granulocytes and 2% monocytes, RBC 250 cells/mcL, glucose less than 10 mg/dl, and protein 458 mg/dl. Ampicillin was stopped. Repeat stool microscopy remained positive for *Strongyloides* spp. Ivermectin dose was increased to 400 mcg/kg daily in 40% ethanol to increase absorption, and albendazole 400 mg q12 hr was also added. On D10, CSF culture reported *E. coli* resistant to PTZ and cefazolin but sensitive to ceftriaxone (CTX) (Table 1). Identification and sensitivity were done by using the MicroScan WalkAway 40 SI system with Negative combo 44 panels. Cefepime was continued. Her mental status did not improve, and the electroencephalogram was reported to be consistent with vegetative state with cerebral dysfunction. Repeat LP after 2 weeks showed WBC 82 cells/mcL with granulocytes 53%, lymphocytes, 46%, and monocytes 1%, RBC 9 cells/mcL, glucose 51 mg/dl, total protein 81 mg/dl, and LDH 192, and Gram staining and culture were negative. Repeated stool exams were negative for larvae. The patient received 14 days of ivermectin and 7 days of albendazole. She did not improve and underwent palliative extubation on D19 and died 10 days later.

## 3. Discussion

Strongyloidiasis is an emerging parasitic infection in developing countries of tropical and subtropical regions with small endemic areas in the temperate region. However, according to recent epidemiological studies, there is some endemicity even in developed countries, including the United States [3]. Epidemiology is complicated by international travel and migration patterns, and global data are considered inadequate [4]. In our case, the patient was born and grew up in Mexico and had a history of multiple travels to Mexico.

The life cycle of this helminthic parasite is unique and complex, with filariform larvae penetrating the skin and ending up in the gut from various routes involving connective tissue, bloodstream, and lungs. Their ability to cause autoinfection is characteristic, which is also the mechanism for hyperinfection. The clinical course is commonly chronic and symptomatically mild, but in immunosuppressed hosts, hyperinfection syndrome characterized by severe disease with high parasitic load and disseminated infection involving internal organs can occur which could be fatal [5]. Diagnosis is difficult and usually late because of variable presentation. In nonendemic settings, a very high index of suspicion is required for diagnosis. In this case, chronic GI symptoms were suggestive, and she had strong risk factors such as the use of abatacept and prednisone and diabetes [6]. No evidence could be found as to whether she was screened for *Strongyloides* spp. before she was started on immunosuppressive therapy, but the screening could have been lifesaving. History of persistent GI symptoms and Gram-negative bacteremia without other sources of infection aroused high suspicion of strongyloidiasis, so we treated her with one dose of ivermectin while waiting for stool microscopy. Our decision to start treatment prior to obtaining diagnostic evidence was based on clinical judgement, and there is no evidence regarding empirical treatment of strongyloidiasis.

Invasive bacterial infections are a well-known complication of strongyloidiasis, which is explained by bacterial translocation on the nematode's surface as it penetrates the intestinal mucosa [7]. According to one study, out of 30 patients with *S. stercoralis*, 16 had invasive infections, including sepsis, meningitis, pneumonia, peritonitis, and endocarditis caused by enteric bacteria and *Candida* spp. [8]. Although pathogenesis is still unclear, high-grade bacteremia is key to meningitis [2]. Bacterial isolates from meningitis associated with strongyloidiasis are mostly the intestinal commensals including *Escherichia coli*, *Streptococcus bovis*, *Klebsiella pneumoniae*, *Lactococcus lactis*, *Streptococcus sanguinis*, *Enterococcus faecium*, and *Staphylococcus warneri* [9–11]. We used broad-spectrum therapy with PTZ and vancomycin for our patient until blood culture reported growth of GNB.

A week after hospitalization, when the patient was moved out of ICU to regular floors and a day after switching from PTZ to cephalexin, the patient had rapid deterioration of mental status and was found to have meningitis from *E. coli* resistant to PTZ and cefazolin but susceptible to CTX. This situation could have resulted from partially treated CNS infection because of ineffective CNS concentration of PTZ, leading to selection of resistant strains or failure to control high inoculum of *E. coli* infection with seeding of the central nervous system. Clinical worsening while the patient was switched from PTZ to cephalexin which was active for less than 24 hours was most likely coincidental than contributory to worsening of the disease. It has been reported that the clinical use of β-lactam/β-lactamase inhibitor combinations results in the selection of point mutants in TEM penicillinases resistant to inhibitors, referred to as inhibitor-resistant TEMs (IRTs) which are generally susceptible to cephalosporins. Strains with complex mutant TEMs (CMTs) that combine ESBL and IRT mutations are resistant to both groups of antibiotics [12]. In this case, *E. coli* isolated from CSF was not an ESBL producer, was resistant to cefazolin, and was sensitive to CTX. A study has reported 4.1% of *E. coli* and 4.4% of *K. pneumoniae* isolates from bloodstream infections with resistance to PTZ and susceptibility to CTX. Most of these patients were hospital inpatients at infection onset and had recently received antibacterial agents, including β-lactam/β-lactamase inhibitor (BL/BLI), and gastrointestinal translocation was the most common presumed source. They have been found to be nonclonal and associated with deleted or dysfunctional porins contributing to the observed resistance pattern [13, 14].

The patient was initially started on PTZ to cover for Gram-negative infection including *Pseudomonas* spp. In a patient presenting with diabetic ketoacidosis with potential abdominal source of infection and without any meningeal signs, concern for meningitis was not significant. PTZ was switched with cephalexin as per the sensitivity report once the patient clinically improved and completed 7 days of intravenous antibiotics. Since there was no suspicion of meningitis, switching to oral therapy seemed appropriate. The optimal duration of antibiotic therapy for bloodstream infections is unknown, and shorter duration of therapy has been demonstrated to be equally effective [15]. With the view of antibiotic stewardship, there was a missed opportunity to de-escalate from PTZ to ceftriaxone once blood culture was reported. Retrospectively switching to ceftriaxone and increasing dose to the meningitic level could have altered the course of disease. When mental status worsened and meningitis was suspected, the patient was started on cefepime and ampicillin. Cefepime was continued even after the sensitivity results because we wanted to suppress the Gram-negative rods in the gut.

Meningitis could have also been caused by reinfection during the continued migration of larvae. There is a strong possibility that clinical worsening and the poor outcome were from the failure to control spread of the larvae rather than the effect of bacterial infection alone. Treatment of invasive bacterial infections is tied with parasite cure because failure in doing so poses risk for recurrent infections [11]. Ivermectin is the drug of choice, but there is no consensus regarding the dosing, and cure rates vary between 55 and 100% [16]. In our case, stool microscopy was still positive for larvae on D7 after 5 days of ivermectin 200 mcg/kg daily dose after which dosing was increased to 400 mcg/kg daily on 40% ethanol to increase absorption, and albendazole was also added.

Limitation of this report is the lack of further testing and characterization of the two *E. coli* isolates which would have provided answers as to how closely they were related and the possible mechanism of the resistance pattern. It is important for the clinical practitioners to be aware of the occurrence of such a resistance pattern and the prevalence in their practice settings. Enteric bacteria and the bowel flora can cause invasive bacterial infections including meningitis during strongyloidiasis, and there should be high suspicion of meningitis in these cases. Antibiotic therapy should be decided taking into account high rates of resistance found among enteric bacteria and the risk of reinfection until the parasitic infection is cured.

## Figures and Tables

**Table 1:** Antibiotic sensitivity reports for *E. coli* isolates (MicroScan WalkAway, NBC44).

Test panel	*E. coli* (blood culture) MIC/KB value (specimen collected on 12/07/2016)	*E. coli* (CSF culture) MIC/KB value (specimen collected on 12/14/2016)
Ampicillin/sulbactam	Resistant (*R*) > 16/8	*R* > 16/8
Amikacin	Sensitive (*S*) ≤ 16	*S* ≤ 16
Ampicillin	*R* > 16	*R* > 16
Aztreonam	*S* ≤ 8	*S* ≤ 8
Ceftriaxone	*S* ≤ 8	*S* ≤ 8
Cefoxitin	*S* ≤ 8	*S* ≤ 8
Cefazolin	*S* ≤ 8	*R* > 16
Ciprofloxacin	*R* > 2	*R* > 2
Ertapenem	*S* ≤ 1	*S* ≤ 1
Gentamicin	*S* ≤ 4	*S* ≤ 4
Imipenem	Not tested	*S* ≤ 4
Piperacillin/tazobactam	*S* ≤ 16	*R* > 64
Trimethoprim/sulfamethoxazole	*R* > 2/38	*R* > 2/38
